# A Non-Invasive Approach to Pulmonary Hypertension

**DOI:** 10.3390/jcm14051473

**Published:** 2025-02-22

**Authors:** Dalma Horvat, Rares Ilie Orzan, Lucia Agoston-Coldea

**Affiliations:** 1 2nd Department of Internal Medicine, Iuliu Hatieganu University of Medicine and Pharmacy, 2-4 Clinicilor, 400006 Cluj-Napoca, Romania; orzanrares@gmail.com (R.I.O.); luciacoldea@yahoo.com (L.A.-C.); 2 2nd Department of Internal Medicine, Emergency County Hospital, 400347 Cluj-Napoca, Romania

**Keywords:** pulmonary hypertension, right ventricle-pulmonary artery unit, non-invasive imaging, cardiac magnetic resonance

## Abstract

Pulmonary hypertension (PH) is a life-threatening cardiopulmonary disease associated with a poor prognosis, with progressive right ventricular (RV) failure being the main cause of death in this vulnerable population. Right heart catheterization remains the gold standard for assessing pulmonary hemodynamics. However, due to its invasive nature, non-invasive imaging methods are gaining increasing interest. Two-dimensional transthoracic echocardiography serves as the primary screening tool for PH and is widely used to estimate its likelihood. Nevertheless, this technique has several limitations, partially addressed through the assistance of a three-dimensional echocardiography. Cardiac magnetic resonance imaging (CMR) provides a comprehensive evaluation of both the morphology and hemodynamics of right ventricle-pulmonary artery unit, offering essential information for diagnosis, prognosis, and therapeutic monitoring. While two-dimensional cardiac CMR enables non-invasive characterization of pulmonary hemodynamics, advances in 4D-flow cardiac CMR allow for a more detailed analysis. These advancements enable the assessment of flow patterns, energetics, wall shear stress and severity, offering a more nuanced understanding of the disease. This review aims to provide an in-depth summary of the current data on advanced non-invasive imaging techniques for PH.

## 1. Introduction

Pulmonary hypertension (PH) is a complex and progressive cardiopulmonary condition, diagnosed based on a resting mean pulmonary arterial pressure (PAPm) exceeding 20 mmHg, as measured by right heart catheterization (RHC). PH is clinically classified into five groups: pulmonary arterial hypertension (PAH), PH caused by left heart diseases, PH caused by lung pathologies or hypoxia, PH secondary to conditions causing pulmonary artery (PA) obstruction, and PH of undetermined or multifactorial origin [1]. The global prevalence of PH is currently estimated at approximately 1%. Left heart disease is the leading cause of PH, followed by lung disease [2]. Among the five groups of PH, PAH has the worst prognosis. Its etiologies include idiopathic and hereditary forms, as well as causes such as toxin or drug exposure, human immunodeficiency virus infection, connective tissue disease, liver disease, schistosomiasis, and congenital heart conditions [3,4]. As a result of progressive remodeling of the pulmonary vasculature, PH causes severe right ventricular (RV) dysfunction and, without treatment, damage to multiple organ systems [4,5].

Despite its limitations, two-dimensional transthoracic echocardiography (TTE) remains a valuable non-invasive screening tool for PH in current clinical practice [6]. Three-dimensional echocardiography (3DE) offers additional advantages by providing a geometrically precise evaluation of the RV. It allows for an accurate assessment of RV function and RV-pulmonary artery (RV-PA) coupling, which is crucial since RV dysfunction is associated with a poor prognosis [7].

Although RHC remains the gold standard for evaluating pulmonary hemodynamics, it is an invasive procedure with potential complications and radiation [8]. In contrast, cardiac magnetic resonance (CMR) is gaining recognition as a promising non-invasive imaging modality for PH. Two-dimensional phase-contrast imaging (2D-PC) is effective for measuring blood flow and evaluating the pulmonary hemodynamics [9]. Moreover, a four-dimensional (4D) flow CMR provides a detailed analysis of blood flow patterns, flow energetics, and wall shear stress (WSS), addressing the limitations of earlier imaging methods [10].

This review aims to present a comprehensive summary of the existing data on the non-invasive evaluation of PH.

## 2. Transthoracic Echocardiography

TTE is widely recognized as the method of choice for assessing the likelihood of PH due to its broad availability, bedside applicability, relatively low costs, and lack of radiation or contrast agent exposure. According to the latest international guidelines, the primary aim of TTE is to estimate the probability of PH by performing a comprehensive evaluation that includes a systolic PA pressure (PAPs) measurement. PAPs is derived using the simplified Bernoulli equation, with the addition of an estimated right atrial pressure [1,11]. Doppler echocardiography can detect a tricuspid regurgitant jet velocity exceeding 2.8 m/s, a finding suggestive of PH. Additional echocardiographic markers, such as RV morphology and function, PA diameter, pulmonary regurgitation velocity in early diastole, acceleration time in the RV outflow tract, inferior vena cava diameter and collapsibility, and right atrial pressure, further refine the likelihood of PH. Based on these findings, the likelihood of PH can be categorized as low, intermediate, or high, guiding the need for further investigations [6,12].

TTE also enables the estimation of PAPm and diastolic pulmonary artery pressures (PAPd) in the presence of pulmonary regurgitation. These parameters are derived using continuous Doppler techniques to measure early diastolic (protodiastolic) and late diastolic (telediastolic) velocities of the pulmonary regurgitation jet, combined with right atrial pressure calculation. Additionally, PAPm can be determined using acceleration time (AT) in the RV outflow tract with the formula: 90 − (0.62×AT-RVOT) [13].

Pulmonary vascular resistance (PVR) can also be evaluated using TTE, demonstrating good correlation with the values obtained by invasive methods [14,15]. Elevated PVR in patients with PH suggest that the underlying pathology is more likely associated with pulmonary vasculature than with left heart disease. The ratio of tricuspid regurgitation velocity (TRV) to the time-velocity integral of the RV outflow tract (TRV/TVI-RVOT) has proven reliable in identifying patients with elevated PVR [13]. Abbas et al. showed that a TRV/TVI-RVOT ratio > 0.275 corresponds to a PVR > 6 Wood units (WU). Additionally, the squared TRV/TVI-RVOT ratio (TRV^2^/TVI-RVOT) offers an even more accurate non-invasive estimation PVR [15]. Chubuchny et al. validated a novel algorithm in a large cohort of patients with heart failure and PH to estimate PA wedge pressure and PVR. This model incorporates multiple echocardiographic parameters, including TRV, left ventricular ejection fraction, RV fractional area change, left atrial volume index, E/e’ mitral ratio, inferior vena cava diameter, PAPm, and cardiac output. This model stratifies patients into PH subgroups: precapillary, isolated postcapillary, or combined (precapillary and postcapillary), with superior diagnostic accuracy compared to the method described by Abbas et al. [15,16].

Although Doppler TTE is a handy and useful imaging tool for PH screening, it also has limitations. Data from the literature indicate that both underestimation and overestimation of PAPs, compared to RHC, can occur [17]. Common pitfalls leading to PAPs underestimation include a severe tricuspid regurgitation, which may cause pressure equalization between the right heart chambers and truncate the tricuspid regurgitation Doppler envelope, as well as advanced lung disease, and minimal tricuspid regurgitation, which can result in an incomplete Doppler spectrum [13,18,19]. Moreover, an overestimation of right atrial pressure, cardiac electrical activity abnormalities, and systemic arterial hypertension are all responsible for the overestimation of PAPs [19,20].

**Figure 1:**
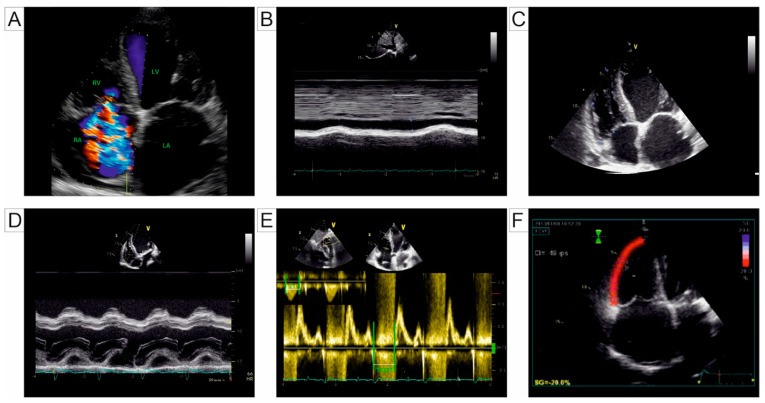
Transthoracic Echocardiography 2D views: (**A**) apical 4-chamber view illustrating severe tricuspid regurgitation assessed through Doppler echocardiography. (**B**) Subcostal view utilized for the management of the inferior vena cava and evaluation of its respirophasic collapsibility, aiding in the assessment of right atrial and PA pressures. (**C**) Apical 4-chamber view at end-diastole in a patient with RV dilation, determined by measuring basal, medial, and longitudinal diameters. (**D**) Apical 4-chamber view with M-mode for estimating lateral tricuspid annulus excursion towards the apex during systole, used as a marker of RV systolic function. (**E**) Pulsed-wave Doppler echocardiography for measuring the RVMPI. (**F**) Speckle-tracking echocardiography at the level of the RV free wall for assessing global RV deformation in a patient with PH. Abbreviations: 2D, two-dimensional; PA, pulmonary artery; RV, right ventricle; RVMPI, right ventricle myocardial performance index; PH, pulmonary hypertension; RA, right atrium; LA, left atrium; LV, left ventricle.

Additional echocardiographic parameters have been proposed to assess the severity of PH, many of which have important prognostic value. These include measurements of RV dimensions at the basal and mid-ventricular levels and along the longitudinal axis, as well as semi-quantitative markers of RV function such as fractional area change (FAC), tricuspid annular plane systolic excursion (TAPSE), and systolic longitudinal contraction velocity (s’) assessed via pulsed tissue Doppler [12,21]. The myocardial performance index (Tei index) is another parameter for RV systolic function, derived either from using pulsed wave Doppler at the tricuspid and pulmonary valves or from tissue Doppler imaging at the lateral tricuspid annulus [13]. Two-dimensional echocardiographic parameters used for PH evaluation are represented in Figure 1.

The 3DE is a promising advancement that addresses the limitations of two-dimensional echocardiography in imaging the RV. It allows for accurate assessment of RV volumes and systolic function in PH patients and demonstrates a strong concordance with CMR, as reported by Li et al. [22]. Furthermore, 3DE enables the evaluation of RV-PA coupling, which is critical for predicting adverse clinical outcomes in precapillary PH. Li et al. demonstrated that RV-PA uncoupling, defined by an RV ejection fraction/PAPs ratio < 0.44, is associated with adverse outcomes. The authors also noted that PA compliance and total pulmonary resistance deteriorate with advancing WHO functional class [23]. Additionally, an RV shape analysis can help differentiate a normal RV from one with pressure overload. Addetia et al. used a curvature analysis to enable a quantitative evaluation of the right heart remodeling, finding that patients with PAH exhibit a more convex septum and a loss of the RV’s bellow-like-action, with persistent convexity of free-wall segments throughout the cardiac cycle [24].

## 3. Cardiac Magnetic Resonance Imaging

CMR is an advanced imaging tool, with significant utility in the morphological and functional assessment of the RV-PA unit. A recent systematic review and meta-analysis underscored that CMR is more than a supplementary imaging tool, confirming its prognostic power in PH. Beyond its ability to predict mortality, metrics related to RV function and volumes are also effective in forecasting clinical worsening [25]. Van der Bruggen et al. demonstrated that at a one-year follow-up CMR parameters could distinguish between low-risk and high-risk PH patients. Integrative models combining functional and imaging parameters provided enhanced prognostic insights [26]. Particularly in individuals with connective tissue diseases, CMR offers superior performance in PH assessment, with mass and volume measurements proving useful for outcome prediction [27].

CMR sequences, including black-blood, steady-state free precession (SSFP), or magnetic resonance angiography (MRA) are all reproducible tools for measuring PA diameters. Among these, cine-SSFP stands out by enabling the measurement of both systolic and diastolic diameters, along with other metrics [28,29]. Reference values for the transverse diameters of the main PA and its branches in adults have been established using 2D-SSFP CMR, showing robust inter-observer agreement [30,31]. Burman et al. further described the variations in PA dimensions based on age and body surface area [31]. Non-invasive assessment of PA distensibility using cine-CMR with the SSFP technique has shown promise in distinguishing idiopathic PH patients with acute vasodilator response from the non-responders, based on minimal and maximal sectional area measurements [32].

Balanced SSFP cine-CMR and black-blood imaging provides high-accuracy regression models using metrics such as the ventricular mass index, interventricular septal angle, and black-blood flow score to enhance PH [33]. Double inversion recovery (DIR) black-blood imaging is particularly valuable for detecting turbulent flow by assessing pulmonary flow artifacts (PFA) in PH patients. This technique grades PFA severity, aiding the PH diagnosis with a sensitivity of 86% and specificity of 85%, as demonstrated by Swift et al. The extent and proximity of the intraluminal smoky signal within the PA tree correlate with PH severity [34].

**Figure 2:**
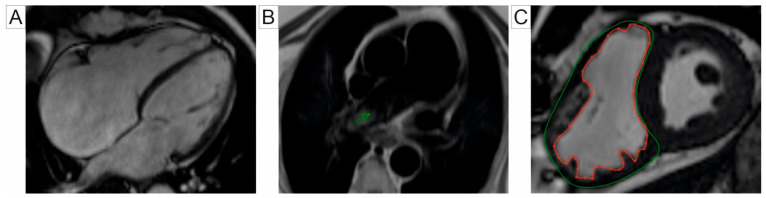
Cardiac magnetic resonance parameters for the assessment of the right ventricle and pulmonary artery: (**A**) Cine-CMR 4-chamber view showing dilated right heart chambers. (**B**) T1-weighted double inversion-recovery black-blood turbo spin-echo images in patient with PH, demonstrating dilatation of the PA with pulmonary flow artifact in the distal right PA (arrow). (**C**) Short-axis CMR image illustrating RV planimetry, involving manual tracing of myocardial boundaries: the epicardial border is marked in green, and the endocardial border is marked in red, measured in end-diastole. Abbreviations: RV, right ventricle; CMR, cardiac magnetic resonance; PA, pulmonary artery; PH, pulmonary hypertension.

CMR is the gold standard for evaluating the right heart function [35,36], a critical determinant of prognosis in PH patients [37]. The SSFP sequence is commonly used for acquiring high-resolution volumetric datasets of the RV due to its superior blood-tissue contrast. RV volumes, mass, and function can be quantified with high intra- and inter-observer consistency and excellent reproducibility across different studies. CMR effectively detects an elevated RV end-diastolic volume, RV dilation, reduced ejection fraction, and RV hypertrophy, which are characteristic of PH [38,39]. Cine-CMR illustrates RV morphological changes, including the flattening and bowing of the interventricular septum (IVS) toward the left ventricle during the cardiac cycle. The degree of IVS curvature correlates with PAPs [40,41]. The RV end-systolic remodeling index (RVESRI), derived from CMR, is calculated as the ratio of RV free wall length to septal height at end-systole. RVESRI is strongly associated with RV remodeling and dysfunction, and an elevated RVESRI correlates with increased PA pressure and afterload. Zhang et al. reported that a RVESRI value greater than 1.35 has excellent diagnostic performance, with a sensitivity and specificity of 97.83% and 83.33%, respectively, for predicting PAPm exceeding 25 mmHg, in patients with chronic thromboembolic pulmonary hypertension (CTEPH) [42]. CMR used for RV-PA evaluation can be seen in Figure 2.

### 3.1. Two-Dimensional and Four-Dimensional-Flow CMR Assessment of Pulmonary Hypertension

**Figure 3:**
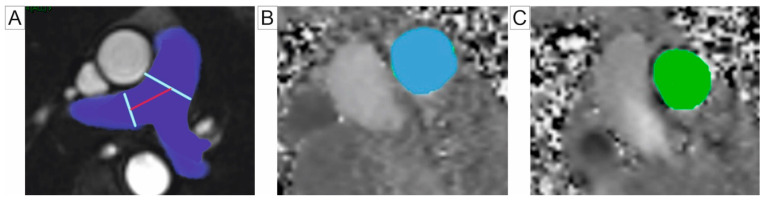
Cardiac magnetic resonance parameters for the assessment of the pulmonary artery: (**A**) axial cine SSFP images showing a cross-section of the MPA and its branches, with measurement planes for blood flow analysis demarcated by blue and red lines. (**B**) Two-dimensional-PC through-plane imaging using CMR, with the flow measurement region in the MPA highlighted by a blue overlay. (**C**) Two-dimensional-PC through-plane imaging using CMR, with the flow measurement region in the RPA, highlighted by a green overlay. Abbreviations: SSFP, steady-state free precession; 2D-PC CMR, two-dimensional phase-contrast cardiac magnetic resonance; MPA, main pulmonary artery; RPA, right pulmonary artery.

Phase-contrast (PC) CMR is a non-invasive imaging modality that quantifies blood flow and assesses haemodynamic changes by measuring blood velocity and volume flow in the PA, thus providing valuable insight into pulmonary circulation and right heart function [9]. The 2D-PC CMR facilitates a non-invasive analysis of PA stiffness through parameters such as pulse wave velocity (PWV). PWV reflects the compliance of the pulmonary vasculature and is typically measured using velocity maps of the PA obtained above the sigmoid valves. The transit time approach, which estimates the duration for a pressure wave to travel between two points in a vessel, is a commonly used method for PWV evaluation. However, its application is often restricted to the proximal PA due to the relatively short length of the vessel [43,44,45]. Bradlow et al. determined standard PWV values in healthy, non-smoking individuals, reporting 2.33 ± 0.44 m/s in the right PA and 2.09 ± 0.64 m/s in the left PA [45]. Combining high temporal resolution imaging with the flow-area method has demonstrated a superior accuracy and reproducibility for PWV measurements in the PA [46]. This methodology has shown particular promise in the pediatric population, as illustrated by Poon et al. [47]. CMR has shown a comparable accuracy to RHC in evaluating proximal PA stiffness, both at rest and during exercise. CMR has been effective in detecting early signs of arterial stiffness that may remain undetectable at rest, making it a valuable tool for an early identification of vascular changes in diseases such as PH [48,49]. The velocity transfer function (VTF), introduced by Gupta et al., represents a novel non-invasive method to assess PA impedance. VTF quantifies the relationship between flow velocity and pressure, serving as a reliable surrogate marker for PA stiffness and resistance [50]. This technique has particular relevance in PH patients with chronic obstructive pulmonary disease, as an increased mean high-frequency modulus (MHFM) of VTF is associated with impaired exercise tolerance and RV remodeling [51]. Although promising, additional validation studies are necessary to confirm the clinical utility of VTF as a diagnostic and prognostic marker. PH evaluation using 2D-PC CMR can be seen in Figure 3.

Four-dimensional flow CMR is an advanced imaging technique that encodes velocity across three spatial dimensions and time. This innovation overcomes the limitations of traditional 2D-PC CMR, echocardiography, as well as computer tomography (CT), which are less effective in accurately measuring blood flow velocities [10]. In addition to providing fundamental 2D hemodynamic data, 4D flow CMR allows advanced visualization tools such as streamlines to depict blood flow patterns (e.g., laminar, helical, or vortical flow) and velocity vectors. It also enables a quantitative analysis with metrics like WSS and flow energetics, which are predominantly used in research settings [10,52,53]. This technique has broad applications, offering valuable insights into energy distribution and blood flow in large vessels and the heart in both healthy individuals and patients with cardiovascular pathologies [10]. While its primary use lies in evaluating congenital heart diseases, 4D flow CMR has also proven beneficial in assessing PH and pulmonary valve disease [52,54]. In addition, its ability to detect early hemodynamic changes makes it particularly useful for patients with connective tissue disease, potentially enabling early diagnosis before the onset of PH [55,56]. Studies have demonstrated a good agreement between 2D and 4D flow CMR, with the latter showing superior accuracy in evaluating pulmonary flow, RV volumes, and function [57,58,59]. Specifically, 4D flow CMR provides more precise flow quantification through pulmonary valve tracking, making it the preferred method for patients with repaired Tetralogy of Fallot [57,58].

In non-diseased individuals, blood flow within the PAs is predominantly laminar, with vortical flow being in a rare occurrence primarily influenced by the unique bifurcation structure of the main pulmonary artery (MPA) [59,60]. Gbinigie et al. demonstrated that flow asymmetry is evident near the peak systole in healthy subjects. Within the MPA, blood flow adopts a helical pattern comprising of two counter-rotating helices. This pattern contributes to the development of a pronounced rightward rotational flow in the right PA (RPA), while the left PA (LPA) exhibits a weaker leftward rotation [61,62]. In addition, the RPA is larger in dimension and exhibits a higher peak stroke velocity when compared to the LPA [61].

**Figure 4:**
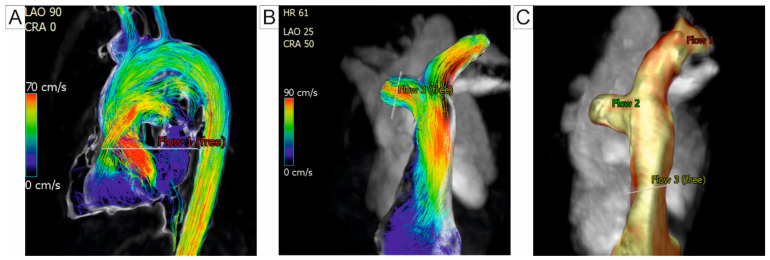
4D-Flow CMR (**A**) Streamlines depicting the instantaneous direction and velocity of blood flow within a vessel or cavity. The color map represents flow velocity, with red indicating higher speeds and blue indicating lower speeds. (**B**) Flow pattern visualization in a patient with PH. (**C**) 3D reconstruction of the PA tree, showing the anatomical vessel geometry and flow regions labeled as Flow 1, Flow 2, and Flow 3 for detailed analysis. Abbreviations: 4D-flow CMR, four-dimensional flow cardiac magnetic resonance; 3D, three-dimensional; PA, pulmonary artery.

In PH, blood flow is typically irregular and turbulent, with disrupted circular patterns. Using the 4D-flow CMR, the visualization of vortex emergence within the PA vasculature is possible, serving as a valuable marker for identifying overt PH. CMR-based measurement of vortical blood flow duration in the PA trunk have been shown to accuratly estimate PAPm in resting states, enabling the non-invasive detection of PH, as suggested by Reiter et al. [63,64]. Their findings indicate that the vortical flow begins to emerge when PAPm exceeds 16 mmHg, with the duration of the vortex increasing proportionally to PAPm [63]. To differentiate the patients with PH from healthy subjects, Schafer et al. quantify helicity and vorticity in the PA. The authors found that helicity significantly decreased in the main pulmonary artery (MPA) and RPA, whereas vorticity decreased only in the RPA. Notably, these parameters showed no significant variability between the different PH subgroups. Among these markers, total helicity emerged as the non-invasive marker with the highest diagnostic precision [65]. Ramos et al. demonstrated that assessing the CMR-derived vortex duration offers superior diagnostic accuracy for tracking the PA pressure compared to the transtricuspidian gradient measurement obtained via a Doppler echocardiography [66]. Another approach to the quantifying vortex flow involves evaluating backward flow components, such as duration, volume flow rate, and cross-sectional area. Kamada et al. evaluated patients with PH due to CTEPH and concluded that these indicators are reliable for evaluating therapeutic outcomes, particularly after a balloon pulmonary angioplasty [67]. Figure 4 illustrates the PH assessment using 4D-flow CMR.

WSS is an emerging and advanced parameter that quantifies the force exerted by blood flow parallel to the vessel [52]. Preclinical research has linked alteration in WSS to abnormalities in elastin and collagen composition and decreased nitric oxide release from pulmonary endothelial cells, which collectively contribute to pulmonary circulation remodeling [68,69,70]. Recently, WSS derived from 4D-flow CMR has proven to be a valuable clinical tool for managing pulmonary vascular diseases, offering unique hemodynamic insights beyond those provided by traditional measurements. Studies have consistently reported a significant decrease in WSS among patients with PH, particularly in advanced disease stages [71,72,73,74]. Absolute WSS values vary based on factors such as vascular diameter, flow velocity, or population heterogeneity. Schafer et al. and Barker et al. reported similar in-plane WSS values in MPA of PH patients, measuring 0.18 ± 0.07 N/m^2^ and 0.19 ± 0.06 N/m^2^, respectively [71,72]. Schafer et al. also identified regional differences in systolic WSS magnitude in the MPA, particularly in the distal right lateral and right anterior segments [71]. Terada et al. analyzed WSS and the index of oscillatory shear (OSI) as markers of flow dynamic abnormalities in secondary PH. Their study revealed that both systolic and mean WSS were significantly lower in disease states and negatively correlated with PA pressures, whereas OSI values were higher and positively correlated with PA pressures [75]. Further supporting the clinical utility of WSS, Ikoma et al. used 4D-flow CMR in patients with connective tissue disease, specifically systemic sclerosis, to detect early changes in pulmonary hemodynamics. They demonstrated the utility of WSS and OSI in identifying early PA abnormalities and proposed additional markers, such as a minimum PA flow and Reynolds number for predicting adverse events in this group of patients [56]. Regardless of age group, decreases in shear hemodynamic indices are strongly associated with vessel dilation and stiffness [76].

PH is a condition that disrupts the entire pulmonary circulation and leads to right heart dysfunction, significantly impacting patient prognosis. The development of the 4D-flow CMR allows advanced assessment of RV function through energetics quantification, facilitating accurate clinical staging of PH patients [9]. Han et al. analyzed two metrics of kinetic energy, the kinetic energy work density and viscous energy loss, and reported significant elevations in these parameters among patients with PAH, compared to healthy controls. These findings highlight the potential of flow energetics as promising imaging markers for distinguishing between patients with and without PH [77]. However, further research is needed to validate these findings and expand their applications.

**Figure 5:**
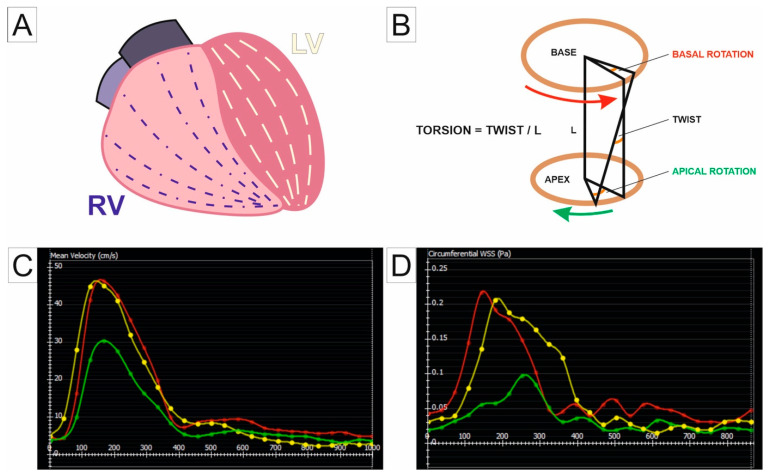
Torsion Imaging. (**A**) Schematic representation of RV subendocardial myofibers arranged in a longitudinal orientation. (**B**) Model illustrating a ventricular torsion, defined as the rotational motion between the base (top) and apex (bottom) during the cardiac cycle. The apex rotates counterclockwise (green arrow), while the base rotates clockwise (red arrow). “Twist” refers to the angular difference in rotation between the apex and base of the heart. Torsion is the twist normalized by the length (L) between the base and apex, describing the rate of twist per unit length of the ventricle. (**C**) Velocity-time curves in the PAs. The vertical axis indicates the mean velocity in cm/s, and the horizontal axis shows time in milliseconds. The curves represent the MPA (red), RPA (green), and LPA (yellow). (**D**) WSS in the PAs. The vertical axis indicates the circumferential WSS in Pa, and the horizontal axis shows time in milliseconds. The color-coded curves correspond to different regions: MPA (red), RPA (green), and LPA (yellow). Abbreviations: RV, right ventricle; LV, left ventricle; WSS, wall shear stress; MPA, main pulmonary artery; RPA, right pulmonary artery; LPA, left pulmonary artery.

Given the poor prognosis of untreated PH, identifying high-risk patients is critical for effective management. Lewis et al. employed a threshold-based approach to improve baseline and follow-up risk stratification, identifying three CMR metrics as significant facilitators in this process: (a) percentage predicted RV end-systolic volume index (>227%), (b) left ventricular end-diastolic volume index (<58 mL/m^2^), and (c) RV ejection fraction (>54%/37–54%/<37%) [78]. A recent study evaluated the accuracy of multilinear principal component analysis and machine learning for predicting 1-year mortality in patients with PAH. This model identified alterations in left ventricular end-diastole and IVS morphology during end-systole as markers associated with the highest mortality risk. Moreover, the study demonstrated that incorporating these findings into clinical risk scores and CMR metrics improved prognostic accuracy compared to traditional methods [79]. Table 1 provides an overview of the key studies highlighting the application of CMR in PH evaluation, and Figure 5 shows torsion imaging of the RV-PA unit.

### 3.2. Myocardial Strain and Right Ventricular Function in Pulmonary Hypertension

Myocardial strain is a unitless measure derived from the disparity in length between two positions, the before and after motion, used to quantify cardiac muscle function. It can be defined using two different approaches. The Lagrangian strain computes displacements at a fixed material point within the myocardium using the deforming myocardium as a reference. Most imaging modalities rely on this approach. The Eulerian strain measures the strain of tissue at a specific spatial location, where spatial coordinates remain constant, while material points undergo continuous changes [81,82]. Given that RV function is now recognized as an independent predictor of morbidity and mortality in individuals with PH, early detection of subclinical RV alterations using advanced myocardial deformation techniques is particularly important in this population. Unlike traditional echocardiographic indicators of RV systolic function, 2D speckle-tracking echocardiography (STE) has emerged as a promising tool. The STE strain demonstrates reduced values in thromboembolic disease and PH, making it a powerful predictor of outcomes in these conditions. Additionally, the STE plays a significant role in the sequential evaluation of RV systolic function [83]. The myocardium undergoes shape changes, including longitudinal, radial, and circumferential strain. Longitudinal strain represents a reduction in length from the base to the apex, and is quantified with negative values. Radial strain describes the deformation towards the center of the RV, indicating myocardial thickening or thinning during the cardiac cycle, quantified positively. Circumferential strain is measured along the RV’s circumferential perimeter, and it typically has negative values and reflects myocardial fiber contraction [84]. When evaluating longitudinal RV strain, two types are identified: RV free-wall longitudinal strain (RVFWLS) that focuses on the free wall of the RV and RV global longitudinal strain (RVGLS) that includes the entire RV. Although there is no universally accepted consensus on normal values, a meta-analysis by Fine et al. proposed a normal range of −27% ± 2% for RVFWLS [85]. A cut-off value between −20% and −21% is often used to identify abnormal RV function [21]. Motoji et al. demonstrated that patients with PAH who had an RVFWLS of less than −19.4% experienced fewer cardiovascular events, compared to those with an RVFWLS of −19.4% or greater [86].

**Figure 6:**
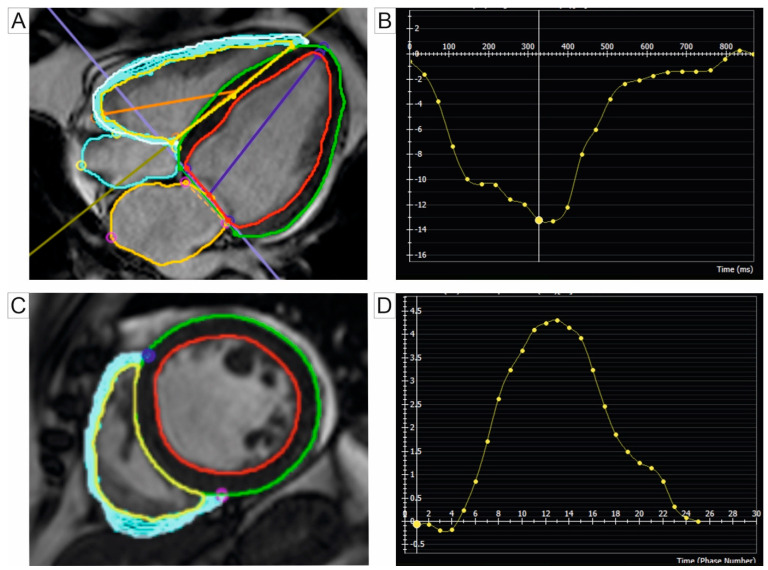
RV strain measurement using CMR FTI. (**A**) FTI map overlaid with longitudinal strain on a 4-chamber cine SSFP image. The green contour delineates the epicardium of the LV, while the red contour outlines the LV endocardium. Similarly, the blue contour represents the epicardium of the RV, and the yellow contour corresponds to the RV endocardium. (**B**) Graph illustrating RV global longitudinal strain, typically represented as negative values, plotted on the vertical axis (e.g., −13%). The horizontal axis represents time in ms. (**C**) FTI map overlaid with radial strain on short-axis cine SSFP image. (**D**) Graph illustrating RV radial strain, typically expressed as positive values, shown on the vertical axis (e.g., 4.3%). The horizontal axis represents time in ms. Abbreviations: FTI, feature tracking imaging; RV, right ventricle; LV, left ventricle; ms, milliseconds; CMR, cardiac magnetic resonance; SSFP, steady-state free precession.

To assess myocardial deformation using CMR, the primary technique is feature tracking (FT), which involves post-processing cine magnetic resonance imaging [87,88]. FT has proven feasible for systemically analyzing RV strain in PH patients [89]. Additional techniques, such as myocardial tagging, displacement encoding with stimulated echoes (DENSE), strain-encoded imaging (SENC), and tissue phase mapping, require specific imaging sequences [88]. SENC outperforms FT in assessing the RV strain due to its superior temporal and spatial resolutions, enabling the capture of all four cardiac chambers in a single heartbeat through rapid SENC [88,90]. In healthy subjects, RV systolic function relies predominantly on longitudinal shortening due to the prevalent longitudinal orientation of myocardial fibers. However, in PH patients, circumferential strain gains greater significance, reflecting adaptation to the disease [90,91]. Table 2 provides a summary of studies on strain imaging of the right cardiac chambers in PH patients, while Figure 6 illustrates the measurement of the RV strain using CMR FTI.

PH and, consequently, RV failure result in significant alterations in the size, function, and pressure of the right atrium (RA), all of which are associated with worse clinical outcomes. Beyond the RV strain, evaluating the RA deformation parameters provides valuable insights into the RV function and can detect subtle changes even when RV ejection fraction remains preserved [96,98]. RA dysfunction, assessed through its reservoir, conduit, and active contraction phases, has been shown to predict mortality and hospital admission rates in individuals with PH [103]. STE and CMR-FT-derived strain analyses have revealed impairments in RA reservoir and conduit function in patients with PH. These parameters are particularly significant, as they reflect the changes in the RV function, even in individuals with preserved RV ejection fraction. Notably, RA phasic performance is primarily influenced by RV lusitropic (diastolic relaxation) dysfunction [96,104,105]. Additionally, Querejeta et al. demonstrated that changes in RA function do not directly correlate with alterations in pulmonary hemodynamics [104].

### 3.3. Myocardial Tissue Characterization

Beyond the conventional assessment of ventricular volumes and ejection fraction, the CMR provides advanced techniques for myocardial tissue characterization, including late gadolinium enhancement (LGE) and T1 mapping. These methods allow for the detection of myocardial fibrosis, edema, and other subtle tissue-level changes that are not evident on standard imaging modalities. Elevated PA pressures increase the RV afterload, triggering a fibrotic remodeling process [106]. Myocardial fibrosis identified through the LGE-CMR typically exhibits a specific distribution at the RV insertion points on the IVS and, in some cases, involves septal regions as well. The extent of LGE has been closely linked with hemodynamic severity and RV dysfunction [107,108,109]. According to Swift et al., the presence of LGE at both the RV insertion points and the septum is linked to poor outcomes [108].

**Figure 7:**
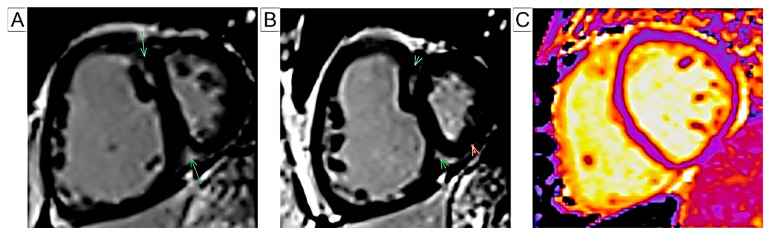
Tissue characterization: (**A**,**B**) LGE CMR image of a short-axis view highlighting fibrosis at the RV insertion points on the IVS (arrows). (**C**) T1-mapping short-axis view. Abbreviations: LGE-CMR, late gadolinium enhancement cardiac magnetic resonance; RV, right ventricle; IVS, interventricular septum.

T1 mapping is a non-invasive CMR technique that evaluates myocardial tissue changes without the use of gadolinium. Elevated T1 values have been observed at the RV insertion points in chronic PH, in both animal and human studies, and are correlated with disease severity [110,111,112]. Saunders et al. demonstrated an association between native T1 values and an increased septal angle [113], while Spruijt et al. reported a correlation with RV remodeling markers [111]. To assess myocardial T1 values at the level of the RV free wall, Asano et al. utilized a customized T1 mapping technique using the modified Look-Locker inversion recovery (MOLLI) sequence. They found that the patients with PAH exhibited elevated T1 values at the RV free wall (RVT1), which showed a strong correlation with the RV performance [114]. Contrary to Saunders et al., the researchers of the latter article identified RVT1 as a predictor of composite clinical outcomes, indicating that native T1 mapping holds prognostic potential in PAH. However, further research is required to validate its utility in clinical practice [114]. Figure 7 illustrates the tissue characterization in PH using different CMR techniques.

Although CMR provides a comprehensive evaluation of patients with PH, it has certain limitations. Compared to other imaging modalities, CMR is less widely available and implies higher costs. Furthermore, despite technical advancements, the long acquisition time and susceptibility to motion artifacts remain significant drawbacks. Patient selection is crucial, as CMR is restricted in those with implanted devices, claustrophobia, renal insufficiency, or allergy to contrast agent [52,115].

## 4. Computer Tomography (CT)

The primary CT techniques for assessing and managing patients with PH include the non-contrast CT (NCCT) and PA CT-angiography (CTA). Both methods allow the identification of PA dilation, estimation of the main PA to ascending aorta (AAo) ratio, and detection of PA tortuosity, calcification, or rapid tapering, findings highly suggestive of PH [115]. In the Framingham Heart Study, reference values for the MPA diameter were established at 27 mm for females and 29 mm for males, with a PA to AAo ratio of 0.9 being associated with PH [116]. According to updated ESC/ERS guidelines, an MPA diameter > 30 mm demonstrates increased specificity and sensitivity in predicting PH, a metric recently validated by Liu et al. [117]. NCCT also provides detailed assessment of the pulmonary parenchyma, aiding in the diagnosing of conditions such as pulmonary arteriovenous malformations, pulmonary capillary hemangiomatosis, and pulmonary veno-occlusive disease, thus contributing to the etiological investigation of PH [115].

CTA is particularly valuable for visualizing the anatomical structure of pulmonary blood vessels and is the gold standard for diagnosing acute pulmonary embolism. It is primarily utilized to detect thrombotic material in central or segmental PAs, with limited sensitivity in subsegmental territories. CTA also reveals vascular abnormalities, including asymmetrical central PAs enlargement with decreased peripheral vessel diameters, eccentrically located calcified thrombi adherent to the vessel wall, and bronchial artery dilation, hallmarks of chronic thromboembolic disease [118]. Additionally, CTA allows for a precise delineation of CTEPH, enabling an experienced multidisciplinary team to evaluate and plan pulmonary endarterectomy for arteries ranging from 10 to 40 mm in diameter [1,119].

Dual energy CT (DECT), an advanced imaging technique that captures images at two different energy levels, is increasingly applied in PH evaluation. DECT provides both qualitative and quantitative insights into pulmonary hemodynamics, as well as parenchymal enhancement [120]. Studies demonstrated a good correlation between DECT lung perfusion assessment and ventilation/perfusion imaging, with a specificity and sensitivity of 99% and 83%, respectively [121]. Another study conducted by Nakazawa et al. reported a specificity of 76% and a sensitivity of 96%, compared to single-photon emission computed tomography [122]. DECT effectively identifies perfusion defects in patients with CTEPH, with distinct vascular characteristics assessable using this technique. A mosaic perfusion pattern contrasting with the localized, wedge-shaped appearance typical of acute PE, is a key indicator [123]. Pontana et al. demonstrated the utility of iodine maps in distinguishing vascular-origin ground-glass opacities from those of bronchioalveolar pathology [124]. Additionally, DECT enables the correlation of PA obstruction severity with perfusion deficit and visualization of calcifications within occlusive clots or PA walls, in cases of prolonged PH. Virtual unenhanced imaging derived from DECT data enhances the detection of systemic collateral circulation and elucidates the relationship between perfusion abnormalities and the extent of systemic collateral supply [123]. DECT provides a comprehensive imaging approach to diagnosis, surgical planning, and follow-up of CTEPH, with perfusion deficits indicating thromboendarterectomy suitability [125,126].

Beyond CTEPH, DECT demonstrates significant benefits in PH characterization of PH. Wook Lee et al. tested its utility in assessing emphysema and regional perfusion in chronic obstructive pulmonary disease [127]. Moreover, Xenon-enhanced DECT (Xe-DECT) enables ventilation mapping to visualize pulmonary function and quantify obstructive ventilation impairment. Sugino et al. demonstrated Xe-DECT potential for differentiating emphysematous from fibrotic lesions in cases of combined pulmonary fibrosis and emphysema [128].

CT scan is an extremely useful imaging method for evaluating patients with PH, particularly in identifying underlying etiologies. The limitations of this technique include its inability to provide data on PA flow, failure to detect early-stage disease, limited capacity to assess right heart function, higher cost compared to echocardiography, and exposure to ionizing radiation [115].

## 5. Conclusions

Recent evidence supports the value of non-invasive cardiovascular imaging methods in the diagnostic work-up, risk stratification, and prognostic assessment of subjects with PH. Furthermore, the significance of CMR in guiding therapeutic decisions continues to grow, though further advancements are needed to optimize its clinical utility. Although the non-invasive evaluation of PH has significantly evolved, an early diagnosis, as well as a personalized risk stratification for these patients remain significant challenges. Future directions focus on eliminating the use of invasive procedures, a key advancement being the use of machine learning tools for diagnostic and management purposes.

## Figures and Tables

**Table 1:** CMR involvement in Pulmonary Hypertension evaluation.

Authors	Year	Purpose	Results
Reiter et al. [64]	2008	To investigate the differences in blood flow patterns in the MPA among patients with manifest PH, latent PH, and healthy subjects, as well as PAPm determination	The presence of vortex flow indicates manifest PH, with t_vortex_ showing a strong correlation with PAPm at rest (r = 0.94 in manifest PH).
Reiter et al. [63]	2015	To evaluate the relationship between invasively measured PAPm and vortex duration derived from PC-CMR	Vortical flow develops in the MPA when PAPm > 16 mmHg, and its duration increases linearly with PAPm; A short-lived vortex (tvortex < 14.3% of the cardiac cycle) indicates borderline PH, while tvortex ≥ 14.3% indicates PH.
Schafer et al. [65]	2017	To evaluate the hemodynamic flow patterns in the PA using 4D-flow CMR and quantify helicity and vorticity in patients with PH	Helicity significantly decreases in the MPA, RPA, and RVOT-MPA unit, whereas vorticity is markedly reduced in the RPA of PH patients; Total helicity proved to have the highest diagnostic accuracy (AUC = 0.937; sensitivity = 90%; specificity = 89.9%; cut-off = 75.2 m/s^2^).
Reiter et al. [80]	2021	To determine the accuracy of the 4D flow CMR for quantifying and predicting changes in PAPm	The AUCs for predicting increases and decreases in PAPm were 0.92 and 0.93; Accuracy, specificity and sensitivity for predicting PAPm increases were 91%, 94% and 85%. For predicting PAPm decreases, these values were 91%, 92%, and 89%; A strong correlation was observed between ΔPAPmMR and ΔPAPm with minimal bias and a SD of differences of 5.1 mmHg in patients with PAPm > 16 mmHg.
Kamada et al. [67]	2022	To quantify the vortex flow in PA of individuals with PH due to CTEPH	Following BPA in CTEPH patients: In the MPA: VFRb, FWHM and area ratio significantly decreased; At the inlet of the PA trunk and main branches, VFR significantly increased; Vessel geometry showed a significant reduction in MPA diameter while blood flow exhibited a significant increase in Re.
Schafer et al. [71]	2016	To evaluate the relationship between MPA-WSS and pulmonary hemodynamics markers and stiffness indices	MPA in-plane WSS was significantly lower in PH patients (0.19 ± 0.07 N/m^2^) compared to controls (0.32 ± 0.08 N/m^2^, *p* = 0.01); In-plane MPA-WSS negatively correlated with mPAP, PVR and E_a_; WSS also significantly correlated with luminal area, stiffness indices, and afterload markers.
Ikoma et al. [56]	2020	To investigate the PA hemodynamics in a population with systemic sclerosis	Maximum WSS was significantly reduced (1.04 ± 0.2 Pa, *p* < 0.05) while OSI was significantly elevated (0.139 ± 0.031 Pa, *p* < 0.01); In SSc patients with adverse composite events, both minimum PA flow and Re were significantly lower, with AUCs of 0.822 (*p* = 0.031) and 0.933 (*p* = 0.004), respectively; Sensitivity and specificity for minimum PA flow were 0.80 and 0.78, while those for Re were 1.00 and 0.89.

Abbreviations: *p* = statistical significance; AUC = area under curve; SD = standard deviation, t_vortex_ = relative duration of vortex formation; PC-CMR = phase-contrast cardiac magnetic resonance; ΔPAPm_MR_ = difference between follow-up and baseline 4D-flow PAPm estimate; CTEPH =chronic thromboembolic pulmonary hypertension; ΔPAPm = difference between follow-up and baseline PAPm; BPA = balloon pulmonary angioplasty; VFR = volume flow rate; FWHM = full width at half maximum; Re = Reynolds number; WSS = wall shear stress; PVR = pulmonary vascular resistance; E_a_ = Effective arterial elastance; OSI = oscillatory shear index; SSc = systemic sclerosis.

**Table 2:** Myocardial strain imaging in subjects with PH.

Reference	Year	*n*	Method	Strain Parameters	Findings
Wang et al. [92]	2023	74 patients with PH	2D-STE	RVFWLS (%) different between groups: mild PH: −21.29 ± 5.39 moderate PH: −17.33 ± 5.25 severe PH: −13.59 ± 5.08	RVFWLS is valuable for early detection of RV dysfunction, demonstrating higher sensitivity compared to TAPSE, S’, and RVFAC; RVFWLS shows enhanced sensitivity for detecting early RV changes in mild PH.
Prieto et al. [93]	2023	22 patients with PH	2D-STE	RVFWLS(apical view): −15% RVFWLS(SCV): −14.5%	A strong correlation exists between SCV and apical measurements of RVFWLS (r = 0.969, *p* < 0.0001, 95% limits of agreement: −3.13 to 3.14); RVFWLS measured via SCV is a reliable and feasible alternative to apical RV strain in patients with poor acoustic windows.
Liu et al. [94]	2023	58 pediatric patients with PH	2D-STE	RVGLS (%): −12.73 ± 5.29 RVFWLS (%): −10.76 ± 7.69	Both RVGLS and RVFWLS strongly correlate with PVR and PAPm (correlation coefficients ranging from 0.6 to 0.8); In pediatric population, longitudinal strain effectively evaluates RV function and aids in assessing disease severity.
Li et al. [95]	2022	41 patients with PAH associated with atrial septal defect (ASD)	CMR-FT	RVGLS (%) in RVEFp PAH-ASD patients: −19.68 ± 2.72	In patients with RVEFp PAH-ASD, GLS > −20% is associated with elevated RVEDP (median: 8 mmHg; range: 6.5–8.25 mmHg) compared to subjects with GLS ≤ −20% (*p* < 0.05); CMR-FT is feasible for RV strain quantification in PAH-ASD population.
Vos et al. [96]	2022	45 patients with pre-capillary PH	CMR-FT	RVGLS (%): −20 ± 6 RVFWLS (%): −25 ± 8 RVGCS (%): −12 ± 5	Higher CS/LS ratio values are observed in precapillary PH; RV function in precapillary PH relies mainly on circumferential myocardial deformation; RV strain is an independent predictor of MACE.
Cao et al. [97]	2022	47 patients with PAH	CMR-TT	RVGLS (%): −11.81 ± 5.46 (−14.7 ± 3.68 in RVEFp and −11.13 ± 5.62 in RVEFr) RVGCS (%): −8.19 ± 3.1 (−10.22 ± 3.26 in RVEFp and −7.71 ± 2.9 in RVEFr) RVGRS (%): 13.18 ± 5.21 (16.85 ± 5.57 in RVEFp and 12.31 ± 4.8 in RVEFr)	CMR-TT demonstrates preferable repetability in GLS, GCS, GRS analysis.
Kallifatidis et al. [98]	2021	36 patients with groups 1 and 4 pre-capillary PH	CMR-FT	RVGLS (%): −20.2 ± 5.3 RVGCS (%): −13.3 RVGRS (%): 24.4 RAGLS (%): −19.9 ± 4.5	An RVGLS cut-off value of −20.62% predicts clinical failure with a sensitivity of 73% (95% CI:48.6–90%) and a specificity of 76.5% (95% CI:49.8–92.1%); Both RA and RV deformation parameters, particularly GLS derived through CMR-FT, aid in prognosis prediction of PH patients.
Evaldsson et al. [99]	2020	55 patients with PH	2D-STE CMR	FWS-ECHO (%): −15.7 ± 4.8 FWS-CMR (%): −20.1 ± 7.7	Moderate correlation is noted for FWS between different modalities (r = 0.656, *p* < 0.001)
Kamide et al. [100]	2020	70 patients with ILD	CMR-FT	RVGLS (%) in patients with PH: −13.3 ± 5.4 RVGLS (%) in patients without PH: −16.9 ± 5.4	RV longitudinal strain with cut-off value of −13.3% predicts mortality with a sensitivity of 100% and specificity of 74%; RVGLS measured by CMR-FT is a valuable non-invasive prognostic indicator for individuals with ILD.
Kemal et al. [101]	2017	91 patients with PH (Group1 and Group 4) on treatment	2D-STE	RVFWS (%): −13.1 ± 6.3	RVFWS corelates well with functional class, 6MWD, NT-proBNP, TAPSE, tricuspid S’, FAC, and RA area; RVFWS with cut-off value of −12.5% predicts clinical RHF onset (sensitivity: 71%, specificity 67%, AUC = 0.692, 95% CI:0.544–0.840, *p* = 0.008) and also cardiovascular adverse events (sensitivity: 54%, specificity: 64%, AUC = 0.630, 95% CI:0.502–0.757, *p* = 0.04).
Muntean et al. [102]	2016	25 children with PAH	2D-STE	RVFW-LpsS (%): −15.6 ± 3.4 (*p* = 0.0001) RVFW-LpsSR (s^−1^): −1.09 ± 0.15 (*p* = 0.0001) Absence of base-to-apex gradient of RVFW-LpsS and RVFW-LpsSR	Mid RVFW-LpsS has predictive value, with a cut-off value of −18.5% predicting clinical worsening with a sensitivity of 91.7% and specificity of 30.8% (AUC = 0.88 ± 0.06, 95% CI:0.75–1.00, *p* = 0.001).

Abbreviations: *n*, number of patients; *p*, statistical significance; r-value, AUC, area under curve; CI, confidence interval; r, correlation coefficient; RVFW-LpsS, right ventricular free wall longitudinal peak systolic strain; RVFW-LpsSR, right ventricular free wall longitudinal peak systolic strain rate; RVGLS, right ventricular global longitudinal strain; RVGCS, right ventricular global circumferential strain; RVGRS, right ventricular global radial strain; ILD, interstitial lung disease; 6MWD, 6 min walking distance; TAPSE, tricuspid annular plane systolic excursion; FAC, fractional area change; NT-proBNP, N-terminal prohormone of brain natriuretic peptide; RA, right atrium; RHF, right heart failure; MACE, major adverse cardiovascular events; RVEFp, right ventricular ejection fraction preserved; RVEFr, right ventricular ejection fraction reduced; RVEDP, right ventricular end diastolic pressure; SCV, subcostal view; FT, feature-tracking; TT, tissue tracking.
